# Neoadjuvant therapy in skin cancer: current evidence and future perspectives

**DOI:** 10.1111/ddg.15968

**Published:** 2026-01-24

**Authors:** Lea Daniello, Johannes Kleemann, Bastian Schilling

**Affiliations:** ^1^ Department of Dermatology University Hospital Goethe University Frankfurt Frankfurt am Main Germany

**Keywords:** melanoma, neoadjuvant, skin cancer

## Abstract

The development of immune checkpoint inhibitors and targeted therapies has fundamentally changed the treatment of cutaneous malignancies, especially in squamous cell carcinoma, melanoma, and Merkel cell carcinoma. The latest neoadjuvant approaches have shown promising results in locally advanced stages.

In squamous cell carcinoma, neoadjuvant PD‐1 blockade with cemiplimab has demonstrated a high pathological response rate. Initial data additionally indicate a lowered risk of recurrence. Even greater progress has been made in melanoma: Neoadjuvant PD‐1 blockade, both alone and in combination with a CTLA‐4 blocker, significantly reduces the risk of recurrence. Both the randomized Phase II SWOG1801 study and the randomized Phase III NADINA study have demonstrated the superiority of the neoadjuvant approach over a pure adjuvant one. The results of the NADINA study showed that a deep pathological response to neoadjuvant therapy allows treatment de‐escalation by omission of adjuvant therapy. Targeted therapies with BRAF‐MEK inhibition also show adequate response rates in BRAF‐mutated melanomas. In Merkel cell carcinoma, the neoadjuvant administration of PD‐1 inhibitors like Nivolumab shows a high response rate and promising survival data. In summary, the data highlight the potential of neoadjuvant therapy in the treatment of locally advanced skin tumors, reducing the risk of recurrence and mortality, as well as providing new opportunities for therapy de‐escalation.

## INTRODUCTION

Skin cancer is one of the most common malignancies worldwide and comprises various entities with individual histological and molecular characteristics. The main types include keratinocyte cancer (KC) and melanoma. KC includes cutaneous squamous cell carcinoma (cuSCC) and basal cell carcinoma, which together account for the majority of skin cancers.^1^ In recent years, increasing evidence suggests that at least a subset of Merkel cell carcinomas (MCC), which represent a rarer entity, may also originate from keratinocytes.^2^ Other rare skin tumors include cutaneous sarcomas, as well as cutaneous B‐cell and T‐cell lymphomas.

In early stages, most skin tumors can be treated with curative intent with surgery. Particularly for KC, radiotherapy also plays an important role in primary, adjuvant, and advanced inoperable settings. In advanced or metastatic cases, targeted therapies (TT) (only in melanoma) and immune checkpoint inhibitors (ICI) are primarily used.^3^, ^4^, ^5^ This article specifically focuses on tumor entities where robust data on neoadjuvant therapy with ICI, TT, and local immunotherapies are available.

With the introduction of ICI and TT, recurrence‐free survival (RFS) and overall survival (OS) have significantly improved in MCC, cuSCC, and melanoma. Increasingly, these systemic therapies are also being investigated in the neoadjuvant setting, although often only in small patient cohorts so far. Larger phase II and III trials on neoadjuvant therapy in melanoma were only first published in 2023/2024. The rationale for the beneficial effect of neoadjuvant therapy is similar across skin cancer entities: it may enable less invasive procedures and potentially eliminate the need for lymph node dissection, thereby reducing morbidity and disfigurement. Moreover, neoadjuvant therapy appears to improve recurrence‐free survival significantly compared to adjuvant therapy. In the case of ICI therapy, this may be due to enhanced T‐cell expansion during the neoadjuvant phase. This expansion is likely triggered by an improved immune response to the tumor that is still present in situ.^6^, ^7^ An additional advantage of neoadjuvant therapy is the early assessment of treatment response, as the histopathological analysis of the resection specimen allows for an objective evaluation of therapeutic efficacy. This can provide prognostic information and support further treatment planning.

**FIGURE 1:**
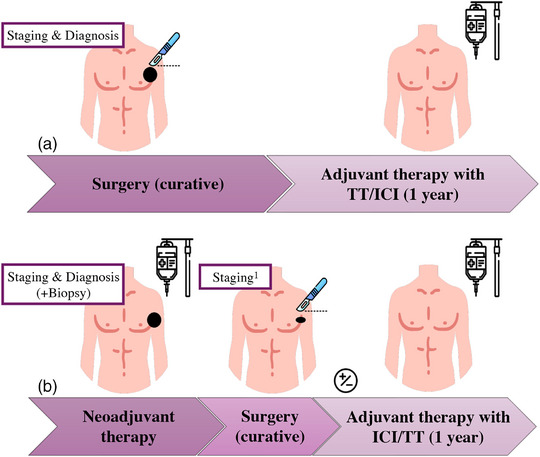
Comparison of treatment concepts (a) adjuvant versus (b) neoadjuvant with optional adjuvant therapy in melanoma. Panel (b) illustrates the possibility of omitting adjuvant therapy depending on the pathological response and the study protocol. *Abbr*.: TT, targeted therapy; ICI, immune checkpoint inhibitor. Icons by MaxIcons and Freepik, all from flaticon.com. ^1^Preoperative imaging to assess radiological response, operability, and to exclude distant metastasis.

The clinical indication for neoadjuvant therapy in malignant skin tumors may be considered in an interdisciplinary context for patients with locoregional or oligometastatic, yet operable, disease (stage II–IV). In addition, standard‐of‐care treatment according to current guidelines should be offered. Depending on the tumor entity, this typically consists of primary surgical resection followed by adjuvant therapy, such as radiotherapy and/or systemic treatment. Figure 1 illustrates a comparison between neoadjuvant and purely adjuvant treatment regimens in melanoma.

The pathological response to neoadjuvant therapy is routinely assessed postoperatively according to the criteria outlined in Table 1.^8^ Based on this assessment, and depending on the study protocol and therapeutic regimen, the appropriate adjuvant therapy is initiated.

**TABLE 1:** Criteria of the International Neoadjuvant Melanoma Consortium for pathological response.

Pathological response	Definition
pCR	No vital tumor cells
npCR/MPR	≤ 10% vital tumor cells
pPR	> 10%–≤ 50% vital tumor cells
pNR	> 50% vital tumor cells

*Abbr*.: pCR, pathological complete response; npCR/MPR, near pathological complete response; pPR, pathological partial response; pNR, pathological non‐response

## NEOADJUVANT THERAPY OF CUTANEOUS SQUAMOUS CELL CARCINOMA

CuSCC is the second most common form of skin cancer, with an annual incidence of 54 new cases per 100,000 men and 26 per 100,000 women in Germany.^9^ In most cases, surgical treatment leads to cure. In patients with a high risk of recurrence, adjuvant radiotherapy may be considered. Approximately 2%–5% of patients develop advanced, unresectable cuSCC, with a 5‐year OS rate of 30%.^10^ Since 2019, the PD‐1 inhibitor cemiplimab has been approved in Europe for the treatment of advanced and metastatic cuSCC.^11^ To date, no neoadjuvant therapy has been approved for cuSCC.

## NEOADJUVANT PD‐1 INHIBITION

Following an initial pilot study, a multicenter phase II trial published in 2022 investigated neoadjuvant treatment with the PD‐1 inhibitor cemiplimab in patients with advanced but resectable stage II–IV (M0) cuSCC.^12^ Seventy‐nine patients received four preoperative doses of cemiplimab 350 mg every 3 weeks (q3w) (Table 2). Postoperatively, patients received adjuvant treatment at the discretion of the investigator, including cemiplimab for up to 48 weeks, radiotherapy, or surveillance. The results showed a pathological complete response (pCR) in 51% and an overall pathological response in 64% of patients. None of the patients with a pCR experienced recurrence within 12 months. Grade 3 toxicities occurred in 4% of patients during the neoadjuvant phase.^13^

Currently, the phase II NEO‐CESQ trial is ongoing, evaluating neoadjuvant and adjuvant cemiplimab in patients with resectable stage III/IV cuSCC. The neoadjuvant protocol consists of two doses of 350 mg cemiplimab q3w, followed by one year of postoperative therapy. Preliminary results from 2023 showed a pathological overall response rate of 52% (12/23 patients), with no grade 3/4 toxicities observed.^14^

## NEOADJUVANT COMBINATION THERAPY: PD‐1 PLUS CTLA‐4 INHIBITION

The Dutch phase II MATISSE trial is currently comparing neoadjuvant treatment with nivolumab monotherapy versus a combination of ipilimumab and nivolumab in patients with stage I–IVa cuSCC. Preliminary data show a major pathological response (MPR) of 40% with nivolumab monotherapy and 53% with the combination therapy. Nine out of 50 patients declined surgery or radiotherapy following neoadjuvant treatment due to a clinical complete remission, which was confirmed by FDG‐PET‐CT. None of these patients experienced recurrence within 12 months.^15^

Follow‐up data, particularly on the safety profiles of the two treatment arms, are still pending. The MATISSE trial suggests that neoadjuvant anti‐PD‐1 therapy may, in some cases, have curative potential in cuSCC and could replace extensive surgical procedures or radiotherapy (Table 2).

**TABLE 2:** Comparison of neoadjuvant treatment regimens in squamous cell carcinoma.

Study	Treatment (neoadjuvant)	n pat.	pCR (%)	MPR (%)	AE G3–4 (%)
NCT04154943	4 x Cemiplimab 350 mg q3w	79	51	13	18
MATISSE	1 x IPI 1mg/kg BW + NIVO 3 mg/kg BW followed by 1 x NIVO 3 mg/kg BW q2w	50	NR	53	12
	2 x NIVO 3 mg/kg BW q2w		NR	40	
NEO‐CESQ	2 x Cemiplimab 350 mg q3w	23	39	8	0

*Abbr*.: AE: adverse events; MPR: major pathological response; n: number; pCR: pathological complete response; IPI: ipilimumab; NIVO: nivolumab; qxw: every x weeks; NR not reported

## NEOADJUVANT THERAPY FOR MELANOMA

The introduction of ICI in 2011, followed shortly thereafter by BRAF/MEK inhibitors, marked the beginning of a new era in melanoma treatment. The median OS for unresectable or metastatic melanoma now reached 6.5 years.^16^ Recently, the indication for PD‐1 blockade has been expanded to include adjuvant therapy for stage II disease. In stage III melanoma, following complete resection, the BRAF/MEK inhibitors dabrafenib/trametinib and the PD‐1 inhibitors nivolumab (also approved in the adjuvant setting for stage IV) and pembrolizumab are authorized, as they significantly reduce the risk of recurrence. However, a survival benefit from adjuvant systemic therapy has not yet been demonstrated.^17^, ^18^, ^19^

## NEOADJUVANT BRAF/MEK INHIBITION WITH DABRAFENIB PLUS TRAMETINIB

One of the first neoadjuvant strategies investigated in melanoma involved the combination of dabrafenib and trametinib. A phase II trial published in 2018 by Amaria et al. compared two treatment arms in patients with stage III–IV (M1a) BRAFV600‐mutant melanoma.^20^ Arm one underwent primary surgery followed by adjuvant standard therapy at the discretion of the investigator. Arm two received 8 weeks of neoadjuvant dabrafenib/trametinib, followed by 44 weeks of postoperative adjuvant therapy. The trial was terminated early due to an interim analysis showing significantly improved event‐free survival (EFS) with neoadjuvant therapy (19.7 vs. 2.9 months) (Table 3).

**TABLE 3:** Comparison of neoadjuvant treatment regimens in melanoma.

Study	Treatment (neoadjuvant)	n pat.	pCR (%)	MPR (%)	AE G3–4 (%)
NCT02231775	Dabrafenib 150 mg + Trametinib 2 mg/d	13	58	NR	61
NeoCombi	12 weeks Dabrafenib 150 mg + Trametinib 2 mg/d	35	49	NR	29
NCT02519322	2x Nivolumab 480 mg + Relatlimab 160 mg q4w	30	57	7	26
Opacin	2x IPI 3 mg/kg BW + NIVO 1 mg/kg BW q3w	9	33	66	90
NCT02519322	NIVO 3 mg/kg BW q2w up to 4x	12	25	NR	8
	3 x IPI 3 mg/kg BW + NIVO 1 mg/kg BW q3w	11	45		73
OpacinNeo	2 x IPI 3 mg/kg BW + NIVO 1 mg/kg BW q3w	30	47	70	40
	2 x IPI 1 mg/kg BW + NIVO 3 mg/kg BW q3w	30	57	64	20
	2 x IPI 3 mg/kg BW followed by 2 x NIVO 3 mg/kg BW	26	23	46	50
PRADO	2 x IPI 1 mg/kg BW + NIVO 3 mg/kg BW q3w	99	49	61	22
*SWOG 1801*	3 x Pembrolizumab 200 mg q3w	154	21	NR	12
*NADINA*	2 x IPI 80 mg + NIVO 240 mg q3w	212	47.2	59	29.7

*Abbr*.: AE, adverse events; MPR, major pathological response; NR, not reported; n, number; pCR, pathological complete response; IPI, ipilimumab; NIVO, nivolumab; qxw, every x weeks

The two most recent and important studies are highlighted in italic.

A subsequent single‐arm phase II trial published in 2019 demonstrated a pathological response in 100% of patients (17 with pCR, 18 with partial pathological response [pPR]). The 2‐year RFS in patients with pCR versus non‐pCR was 63% vs. 24% – notably inferior compared to immunotherapy‐based regimens.^21^ The NeoTrio trial is currently investigating a combination of BRAF/MEK inhibitors with pembrolizumab in patients with stage III BRAF‐mutant melanoma. Preliminary data suggest improved pCR rates with concurrent administration compared to sequential administration or pembrolizumab monotherapy. However, toxicity was higher with the concurrent approach (grade 3/4 toxicities: 55% vs. 25%).^22^

## NEOADJUVANT PD‐1 INHIBITION WITH PEMBROLIZUMAB

In the first study evaluating neoadjuvant pembrolizumab, 30% of patients with stage III–IV disease achieved a pCR or MPR after just one dose. All patients with a pathological response remained recurrence‐free after a median follow‐up of 25 months.^23^

The first large‐scale randomized study of neoadjuvant therapy in resectable stage III/IV melanoma, SWOG1801, was published in 2023.^24^ This phase II trial included 313 patients from 90 U.S. centers and compared neoadjuvant plus adjuvant pembrolizumab to adjuvant therapy alone. In the experimental arm, patients received three neoadjuvant doses of pembrolizumab (200 mg q3w), followed by surgery and 15 additional doses postoperatively. In the control arm, patients underwent immediate surgery followed by 18 adjuvant doses, resulting in the same total number of treatments.

The primary endpoint was EFS, with events defined as recurrence post‐surgery, progression of known metastases, toxicity‐related treatment delays, delayed initiation of adjuvant therapy, or death. After a median follow‐up of 14.7 months, the 2‐year EFS rate was significantly higher in the neoadjuvant group (72% vs. 49%). Toxicity rates were comparable (≥ grade 3/4 toxicities: 12% vs. 14%). Pathologically, 21% of patients achieved a pCR. Imaging (RECIST 1.1 criteria) showed a complete response in 6% and a partial response in 41% of patients after neoadjuvant therapy.

## IPILIMUMAB PLUS NIVOLUMAB

Several major studies on the neoadjuvant combination of ipilimumab and nivolumab have recently been published (Table 3). In a phase II study by Amaria et al., 23 patients received either four cycles of nivolumab (3 mg/kg body weight [BW] every 2 weeks) or three cycles of combination therapy with ipilimumab (3 mg/kg BW) and nivolumab (1 mg/kg BW) every 3 weeks.^25^ The objective response rate (ORR) by RECIST was 25% in the monotherapy group and 73% in the combination group, while the pCR rates were 25% and 45%, respectively. After a median follow‐up of 15.6 months, the combination arm showed improved RFS, but also a higher incidence of ≥ grade 3 toxicities (73% vs. 8%).

Simultaneously, the OpACIN trial was published, a prospective randomized phase Ib/II study in which patients with palpable stage III melanoma received two doses of ipilimumab (3 mg/kg BW) and nivolumab (1 mg/kg BW) in both neoadjuvant and adjuvant settings.^6^ The control arm received four cycles of the same combination therapy in the adjuvant setting only. Although high efficacy was observed (pathological response: 78%), 90% of patients experienced grade 3/4 toxicities. This prompted the subsequent phase II OpACIN‐neo trial, which evaluated optimized dosing regimens.^26^ The best balance between efficacy and toxicity was achieved with a “flipped‐dose” schedule: two doses of ipilimumab (1 mg/kg BW) and nivolumab (3 mg/kg BW) every 3 weeks. This regimen resulted in a 47% pCR rate with only 20% grade 3/4 toxicities. The 3‐year RFS and OS rates for the entire cohort were 82% and 92%, respectively. Among patients with a pCR, RFS was 95%, compared to 37% in those with pathological non‐response (pNR).^27^ A discrepancy between radiological and pathological response, noted in later studies as well, highlighted the higher reliability of pathological response.^28^ Thus, pathological response has emerged as a predictive biomarker for clinical outcomes in melanoma.

In 2022, the OpACIN‐neo data was expanded with the PRADO cohort, in which 99 patients received the optimized combination therapy.^29^ This extension study evaluated the correlation between pathological response in an index lymph node (ILN) and RFS. The ILN was defined as the largest lymph node metastasis at baseline, marked with a clip and removed after two cycles of therapy. In cases of MPR or pCR, therapeutic lymph node dissection (TLND) was omitted; in pPR, TLND was performed; and in pNR, additional adjuvant systemic therapy was given. After 24 months, patients with MPR/pCR had an RFS of 93%, suggesting that omitting TLND in this subgroup may be safe. However, randomized trials are still needed to confirm this de‐escalation strategy for all MPR/pCR cases in the ILN.

In 2024 the NADINA trial, the largest and first phase III study on neoadjuvant therapy in melanoma, was published, marking a milestone in melanoma management.^30^ A total of 423 patients with stage III melanoma and at least one resectable lymph node metastasis were included. Up to three in‐transit metastases were allowed, as neoadjuvant therapy has been shown to be effective in these patients as well.^31^, ^32^ In Arm A, patients received two cycles of fixed‐dose “flipped” ipilimumab (80 mg) plus nivolumab (240 mg) every 3 weeks, followed by TLND. If MPR/pCR was achieved, no adjuvant therapy was given; otherwise, twelve cycles of nivolumab or, in BRAFV600E/K‐mutated patients, dabrafenib plus trametinib (for 46 weeks) were administered. In Arm B, patients underwent surgery followed by twelve cycles of adjuvant nivolumab. Radiotherapy was permitted in both arms except in cases of MPR/pCR. The primary endpoint was EFS, defined as progression to unresectable disease, recurrence, or death due to disease or treatment. After 12 months, the neoadjuvant group showed significantly better EFS (83.7% vs. 57.2%, p < 0.001). A total of 59% achieved MPR or better, thereby avoiding TLND and adjuvant systemic therapy. RFS was 95.1% in those with MPR and 76% in those with partial pathological response. Grade 3/4 toxicities occurred in 29.7% (neoadjuvant) and 14.7% (adjuvant) of patients. These findings underscore the efficacy of neoadjuvant therapy and its potential to enable treatment de‐escalation.

## ADDITIONAL SUBSTANCES

### T‐VEC

Talimogene laherparepvec (T‐VEC) is an oncolytic viral therapy based on herpes simplex virus type 1, administered intralesionally. It is approved for unresectable stage III/IV (M1a) melanoma. A phase II study compared surgery alone with neoadjuvant T‐VEC. The neoadjuvant arm achieved a 2‐year RFS of 29.5%, compared to 16.5% in the surgical arm. 17.1% of patients in the neoadjuvant group achieved a pCR.^33^ An ongoing study is investigating the neoadjuvant combination of T‐VEC and nivolumab.^34^

### Daromun

Daromun is an intralesional immunotherapy using antibody‐cytokine fusions L19IL2 and L19TNF, delivering interleukin (IL)‐2 and tumor necrosis factor (TNF)‐α directly to the tumor, inducing local inflammation and enhancing systemic immune response.^35^ L19 ensures prolonged retention of IL‐2 at the target site.^36^ A phase II study demonstrated efficacy in stage IIIC–IV melanoma, leading to the ongoing PIVOTAL phase III study for neoadjuvant application. In this study, neoadjuvant therapy (13 million IU L19IL2 and 400 µg L19TNF over 4 weeks) was compared with surgery in patients with skin and/or lymph node metastases in resectable stage III. Preliminary data show a median RFS of 16.7 months in the neoadjuvant arm versus 6.9 months in the surgical arm, with a pCR rate of 21%.^35^ The *European Medicines Agency* (EMA) has been petitioned for approval based on this data for resectable stage IIIB–D melanoma, including previously treated patients.^37^ Daromun could become the first approved neoadjuvant melanoma therapy.

### Pembrolizumab plus vibostolimab or gebasaxturev

A phase I/II study published in 2025 investigated the neoadjuvant administration of pembrolizumab combined with either the anti‐TIGIT (T‐cell immunoreceptor with Ig and immunoreceptor tyrosine‐based inhibitory motif domains) antibody vibostolimab or the oncolytic virus gebasaxturev.^38^ TIGIT is a receptor which plays a role in suppressing innate and adaptive immunity. Preclinically, vibostolimab combined with PD‐1 inhibitors showed inhibition of tumor growth. Gebasaxturev is a bioselected, genetically unmodified coxsackievirus, which leads to tumor lysis upon infection and simultaneously aims to enhance tumor infiltration by CD8‐positive T cells.^39^ The study compared three neoadjuvant arms: 2 x pembrolizumab 200 mg plus vibostolimab 200 mg every 3 weeks, versus 1 x pembrolizumab 400 mg plus up to five intralesional injections of gebasaxturev followed by eight cycles of adjuvant pembrolizumab 400 mg, versus 1 x pembrolizumab 400 mg. Pathological response rates were 81%, 52%, and 73%, respectively. The 18‐month EFS rates were 81%, 72%, and 80%. Grade 3/4 toxicity rates were 15%, 40%, and 27%, respectively. Neither vibostolimab nor gebasaxturev is globally approved.

### Nivolumab plus relatlimab

The combination of nivolumab and the anti‐LAG3 antibody relatlimab received approval in 2022 for unresectable or metastatic melanoma. Relatlimab is a human monoclonal antibody that blocks LAG‐3, thereby reducing LAG‐3‐mediated immune suppression and promoting T‐cell proliferation and activation.^40^ In 2022, the first phase II study on neoadjuvant therapy was published, in which patients received neoadjuvant nivolumab twice (480 mg) plus relatlimab (160 mg) every 4 weeks, followed by ten adjuvant cycles.^40^ 57% achieved a pCR, 70% a pathological overall response. After 2 years, the RFS was 92% (responders) and 55% (non‐responders). No severe adverse events occurred in the neoadjuvant phase; 26% developed grade 3/4 toxicity in the adjuvant phase.

### Neoadjuvant therapy in Merkel cell carcinoma

Merkel cell carcinoma is rare but increasing in prevalence.^41^ In up to 80% of cases, the Merkel cell polyomavirus is integrated; alternatively, or additionally, photocarciogenesis can contribute to its development.^42^ Surgery is standard of care in early stages but associated with high recurrence rates and a 5‐year survival rate of 50%–60%.^43^ MCC responds well to PD‐1/PD‐L1 checkpoint inhibitors, as demonstrated in a single‐arm phase II study, leading to the European approval of avelumab (PD‐L1 inhibitor) in stage IV in 2017 (ORR 33%, OS 12.6 months).^44^ In the USA, pembrolizumab is approved for MCC in stage IV based on positive results from a phase II study (response rate 56%).^45^ Due to high recurrence rates in stages III–IV, the phase II ADMEC‐O study investigated adjuvant nivolumab therapy versus observation, with an absolute risk reduction of 9%.^46^ Recently, initial data on neoadjuvant therapy for MCC were published. However, due to the rareness of MCC, the study landscape is limited. The phase I/II CHECKMATE‐358 study investigated neoadjuvant nivolumab (2 x 240 mg every 2 weeks) in 39 patients with resectable MCC (stage IIA–IV), without a fixed adjuvant therapy.^47^ Results were promising: 47% achieved a pCR, 15.4% a major pathological response, and 87.9% a radiological tumor reduction. Similar to melanoma, where 58% of patients with radiological stable disease showed pCR or partial response, pathological response often exceeded imaging results.^28^ After 20 months of follow‐up, median RFS and overall survival were not reached. The RFS was significantly higher in pCR patients (100% vs. 59.6% after 12 months).

## UNANSWERED QUESTIONS AND FUTURE PERSPECTIVES

The data above demonstrate that neoadjuvant therapy in cuSCC, melanoma, and MCC holds significant potential, particularly concerning EFS and RFS. The most likely explanation for the superior effect of neoadjuvant versus adjuvant therapy is that preoperative treatment induces a stronger, more diverse T‐cell response, as the entire tumor is present, thereby activating a broader repertoire of T‐cells.^30^ The randomized phase III study NADINA and pooled smaller studies show that combining PD‐1 blockade with CTLA‐4 inhibition enhances efficacy.^25^, ^48^, ^49^ Ipilimumab expands the tumor‐specific T‐cell repertoire and could thereby improve the antitumor immune response.^50^

While the above studies indicate a significant benefit of neoadjuvant therapy, it remains unclear which patient groups benefit most. In melanoma, only a few patients with resectable single metastases in stage IV were included, which questions the relevance of the data in this therapeutic situation. Also, patients in stage III with extensive primary tumors, satellite, or in‐transit metastases without additional lymph node involvement were underrepresented. Smaller studies, however, show comparable responses to neoadjuvant therapy in synchronous primary tumors or local skin metastases.^6^, ^28^, ^32^ In cuSCC, it should be noted that both in the cemiplimab study by Gross et al. and in the MATISSE study, many patients with T1–T2 stage were included, which skews the EFS data, as these patients often show a favorable course. Furthermore, none of the studies included immunosuppressed patients, who are considered high‐risk according to guidelines.^51^ The exclusion was likely based on retrospective data indicating lower efficacy of ICIs, or due to the risk of triggering the underlying disease or transplant rejection through the use of ICIs.^52^, ^53^, ^54^

In melanoma and cuSCC, it is unclear who benefits from adjuvant therapy after neoadjuvant treatment. The NADINA study showed that patients with a pCR or MPR have excellent RFS even without adjuvant therapy. The SWOG1801 study found similarly good RFS data in patients who received both therapies. To optimize therapy, it would be important to clarify for which patients the sole neoadjuvant therapy with three doses of pembrolizumab is sufficient. Patients with pCR show the best RFS in all skin tumors, as confirmed by a pooled melanoma analysis: after 2 years, RFS was 89% for pCR/MPR patients versus 50% without pCR. Thus pCR could serve as a criterion for therapy de‐escalation.^49^

It remains unclear whether continuing therapy in cases of pathological non‐response (pNR) is sensible, since an “ineffective” therapy would be continued. The histological pattern of pNR could serve as a discriminator.^55^ After the MATISSE study, it is unclear whether preoperative PD‐1 therapy in cuSCC is always neoadjuvant, as an individual curative approach without surgery can also be an option, making the term “neoadjuvant” obsolete. Nine patients without surgery and adjuvant therapy showed a clinical complete remission after one year and an RFS of 100%, suggesting that resection is not always necessary. For MCC, meaningful data on the general indication for adjuvant therapy are lacking; however, patients without pathological response after neoadjuvant therapy in the cited study had early recurrences, indicating the need for individual adjuvant therapy, be it after neoadjuvant treatment or only as adjuvant therapy.

The discrepancy in scores for pathological response complicates the interpretation of study data. Earlier studies defined pCR as complete absence, pPR as ≤ 50%, and pNR as > 50% vital tumor cells. Newer studies introduced the terms MPR or ncPR, defined as 0–≤ 10% vital tumor cells, limiting comparability. Fortunately, the *International Neoadjuvant Melanoma Consortium* published consensus categories in 2018, which should be used in studies and clinical practice.^8^ Nevertheless, clear training of pathologists in the respective centers is required to assess the specimen according to the specifications, which can often be a problem in everyday clinical practice.

As with any ICI therapy, the issue of therapy‐associated side effects arises in neoadjuvant therapy. In studies on neoadjuvant therapy in skin cancer, 10%–60% of patients experienced higher‐grade immune‐mediated side effects. Toxicities can lead to delays in surgery and a higher risk of postoperative complications, especially when corticosteroid therapies are necessary. However, the NADINA and SWOG1801 studies showed positive results: in the NADINA study, surgery had to be canceled in only 1% of patients (n = 3), and in the SWOG study, in only one patient due to toxicities.^24^, ^30^ Nevertheless, there are delays in surgery due to neoadjuvant therapy. Even though only a few patients experienced progression during therapy (2% in NADINA, 7.7% in SWOG), all patients should be informed about the risk of tumor progression that could lead to inoperability.^24^, ^30^

One of the biggest obstacles in clinical practice is the lack of approval for neoadjuvant melanoma therapies in Europe. Additionally, in Germany, reimbursement is not automatically granted. In Australia, Italy, and other countries, authorities have agreed to cover the costs for the SWOG1801 regimen. In Germany, reimbursement for neoadjuvant therapies following the SWOG1801 or NADINA regimens must be clarified with the insurance providers beforehand. Due to prospective, randomized data, there is a valid data foundation that should be referenced. For squamous cell carcinoma and Merkel cell carcinoma, the available data is limited to single‐arm studies, posing a greater challenge for reimbursement applications and highlighting the need for additional studies to strengthen the supporting evidence.

A further issue in clinical practice is appointment coordination. For neoadjuvant care, close coordination of infusion appointments, staging examinations, and surgery dates is essential.

In addition to the neoadjuvant treatment approaches for skin cancer already discussed, further clinical studies are in the recruitment process. One example is the phase II melanoma study IOB‐032, which investigates the safety and efficacy of a combination of the cancer vaccine IO102‐IO103 and pembrolizumab as a neoadjuvant and adjuvant therapy in patients with resectable melanoma.^56^ As mentioned earlier, many of the other discussed studies above are also still ongoing, and more comprehensive evaluations and results are expected in the near future, potentially further underlining the importance of neoadjuvant therapy.

## CONCLUSIONS

Neoadjuvant therapy in skin cancer, particularly melanoma, shows great promise; however, questions regarding patient selection and optimal treatment regimens remain unresolved. Further research is needed to refine therapeutic strategies and maximize patient benefit. Nevertheless, neoadjuvant therapy should already be recommended for all melanoma patients with clinically detectable lymph node metastases in stage III and offered to those with operable stage IV disease, as current data demonstrate a significant improvement in disease outcomes compared to standard treatment approaches.

## CONFLICT OF INTEREST STATEMENT

J.K. has received personal honoraria from Bristol‐Myers Squibb, Sanofi, SUN Pharma, and Amgen for participation in advisory boards or speaking engagements, as well as travel support from Pierre Fabre Pharma. B.S. has received personal honoraria from SUN Pharma, Immunocore, Bristol‐Myers Squibb, Sanofi, Novartis, Regeneron, Pierre Fabre Pharma, and Blueprint Medicines for participation in advisory boards or speaking engagements. L.D. declares no conflict of interest.
